# Effectiveness of a website and mobile phone based physical activity and nutrition intervention for middle-aged males: Trial protocol and baseline findings of the ManUp Study

**DOI:** 10.1186/1471-2458-12-656

**Published:** 2012-08-15

**Authors:** Mitch J Duncan, Corneel Vandelanotte, Richard R Rosenkranz, Cristina M Caperchione, Hang Ding, Marcus Ellison, Emma S George, Cindy Hooker, Mohan Karunanithi, Gregory S Kolt, Anthony Maeder, Manny Noakes, Rhys Tague, Pennie Taylor, Pierre Viljoen, W Kerry Mummery

**Affiliations:** 1 Central Queensland University, Institute for Health and Social Science Research, Centre for Physical Activity Studies, Bruce Highway, Rockhampton, QLD 4700, Australia; 2 Kansas State University, Department of Human Nutrition, , Manhattan, Kansas, 66506, United States; 3 University of British Columbia, School of Health and Exercise Sciences, Kelowna, British Columbia, V1V 1V7, Canada; 4 CSIRO, The Australian eHealth Research Centre, ICT Centre, Level 5, UQ Health Sciences Building 901/16, Royal Brisbane and Women's Hospital, Herston, QLD, 4029, Australia; 5 University of Western Sydney, School of Science and Health, Locked Bag 1797, Penrith, NSW 2751, Australia; 6 University of Western Sydney, School of Computing, Engineering and Mathematics, Tele-Health Research and Innovation Laboratory, Narellan Road, Campbelltown, NSW, 2560, Australia; 7 CSIRO, Food and Nutritional Sciences, PO Box 10041, Adelaide, BC, 5000, Australia; 8 Central Queensland University, Boundary Road, Mackay, QLD, 4740, Australia; 9 University of Alberta, Faculty of Physical Education and Recreation, Edmonton, Alberta, T6G 2H9, Canada

**Keywords:** Physical activity, Nutrition, Males, Website, Internet, Online, Mobile phone, Self-monitoring, Literacy, Intervention

## Abstract

**Background:**

Compared to females, males experience higher rates of chronic disease and mortality, yet few health promotion initiatives are specifically aimed at men. Therefore, the aim of the ManUp Study is to examine the effectiveness of an IT-based intervention to increase the physical activity and nutrition behaviour and literacy in middle-aged males (aged 35–54 years).

**Method/Design:**

The study design was a two-arm randomised controlled trial, having an IT-based (applying website and mobile phones) and a print-based intervention arm, to deliver intervention materials and to promote self-monitoring of physical activity and nutrition behaviours. Participants (n = 317) were randomised on a 2:1 ratio in favour of the IT-based intervention arm. Both intervention arms completed assessments at baseline, 3, and 9 months. All participants completed self-report assessments of physical activity, sitting time, nutrition behaviours, physical activity and nutrition literacy, perceived health status and socio-demographic characteristics. A randomly selected sub-sample in the IT-based (n = 61) and print-based (n = 30) intervention arms completed objective measures of height, weight, waist circumference, and physical activity as measured by accelerometer (Actigraph GT3X). The average age of participants in the IT-based and print-based intervention arm was 44.2 and 43.8 years respectively. The majority of participants were employed in professional occupations (IT-based 57.6%, Print-based 54.2%) and were overweight or obese (IT-based 90.8%, Print-based 87.3%). At baseline a lower proportion of participants in the IT-based (70.2%) group agreed that 30 minutes of physical activity each day is enough to improve health compared to the print-based (82.3%) group (*p* = .026). The IT-based group consumed a significantly lower number of serves of red meat in the previous week, compared to the print-based group (*p* = .017). No other significant between-group differences were observed at baseline.

**Discussion:**

The ManUp Study will examine the effectiveness of an IT-based approach to improve physical activity and nutrition behaviour and literacy. Study outcomes will provide much needed information on the efficacy of this approach in middle aged males, which is important due to the large proportions of males at risk, and the potential reach of IT-based interventions.

**Trial registration:**

ACTRN12611000081910

## Background

Reducing disease and the prevalence of avoidable risk factors associated with poor health in males is attracting considerable attention within the public health agenda. This is evidenced in both developed and developing countries by the advancement of several recent policy initiatives and other strategies dedicated to improving the health of males [1,2]. These initiatives are based on a recognition that, compared to females, males experience higher rates of overall premature mortality, cardiovascular disease related mortality, suicide, diabetes, and obesity [2,3]. These health outcomes are linked to males’ high levels of behavioural risk factors including high levels of physical inactivity, poor nutrition behaviours, high risk drinking and smoking [2,3].

Improving the physical activity levels of males is vital to improving their health, as physical inactivity is one of the most significant risk factors for the leading causes of preventable death and burden of disease in Australian males [2]. Physical inactivity is characterized by high levels of sedentary behaviours, such as sitting, and/or low levels of participation in moderate-to-vigorous intensity physical activity, such as walking. Australian surveillance data indicate that males report more than 3 hours of occupational sitting, and 5.5 hours of total sitting per day [4,5]. Although approximately 53% of males report engaging in sufficient levels of physical activity for health benefit, the proportion of males engaging in sufficient physical activity has not increased in recent years [6].

Improving nutrition behaviours is similarly important for improving male health, as poor nutrition behaviours also rank highly among risk factors for the leading causes of preventable death and disease in Australian males [2]. Poor nutrition behaviours are characterized by low consumption of fruits, fibre, vegetables, and fish, and high consumption of saturated fat and alcohol. Fruit and vegetable consumption is low in males, with fewer than 6.5% of males consuming recommended daily serves of fruit and vegetables [7,8] and 15% reporting that they consumed alcohol at high-risk levels [9]. Furthermore, approximately half of adult males consume full-cream milk on a usual basis and takeaway food at least once per week, which is indicative of high saturated fat intake [10].

Despite ample evidence of demonstrated need for improvement in health and related behavioural risk factors, males are frequently underrepresented in health-promotion and chronic disease prevention intervention efforts [11,12], and report that the interventions that are frequently adopted by females do not appeal to them [13]. Thus, there is a need to develop interventions that specifically target physical activity and nutrition behaviours of males in a way that is tailored to their preferences. The format of the messages, presentation of information delivered, mode of information delivery, and methods to engage participants are key intervention strategies than can be tailored to males. Website-delivered interventions can be tailored to specific user preferences and needs, are viewed favourably by males, and can positively alter levels of overweight, physical activity, and selected dietary behaviours [12,14,15]. The accessibility of such website-delivered interventions may also be more appealing to males who cannot attend face-to-face interventions due to clashes with work, family responsibilities, and other schedules [16]. Additionally the capacity of website-delivered interventions to be accessed by large numbers of males in need of health behaviour change is beneficial. The provision of educational materials, goal setting strategies, and tools to promote self-monitoring have been shown to be effective methods to change physical activity and nutrition behaviours [12,17-19]. Our formative research on these issues demonstrated that males are somewhat knowledgeable on the levels of physical activity and healthy eating required to promote health, but that they also want information on these topics provided in a clear and simple way to avoid confusion associated with multiple media campaigns and messages [20,21]. Tailoring health promotion messages in this way is important as the materials not only need to provide information in an ‘easily digestible’ format for males, but must also empower males to use this information to positively change their behaviours [22]. This process of applying knowledge to change behaviour, or health literacy, plays an important role in health promotion and is relatively low in many populations [22]. Males also acknowledge the importance of self-monitoring behaviours, but want this to be completed in a time-efficient and user-friendly manner [21]. As such, website-delivered interventions that incorporate these characteristics and features may be useful to positively change physical activity and nutrition behaviours.

Self-monitoring of health behaviour is a common behaviour change strategy implemented in many health promotion interventions [23], and is positively associated with greater behaviour change across a range of health behaviours [17,18]. Mobile (cell) phones that store data locally and/or connect to the internet offer participants a modern and technologically sophisticated way to self-monitor behaviour, may offer greater convenience when compared to more traditional self-monitoring methods, and allow transfer of data to a website that contains other intervention materials and components [21]. However, the use of websites and/or mobile phones, to self-monitor physical activity or nutrition behaviours, specifically targeted at males, remains largely untested. This manuscript describes the rationale, design, and baseline findings from the ManUp Study, which aims to examine the effectiveness of an information technology (IT) based intervention that uses both websites and mobile phones to improve the physical activity and nutrition behaviour and literacy in middle-aged males.

## Methods/Design

The ManUp Study design is a two-arm randomised controlled trial with assessment points at baseline, three, and nine months. One intervention arm used an IT-based approach (website and mobile phones) to deliver physical activity and healthy eating promotion materials, and to promote self-monitoring of these behaviours. The second intervention arm used a more conventional print-based approach to deliver the same health promotion materials. Both intervention arms provided participants with the same materials and ability to self-monitor their behaviours, although the IT-based intervention additionally provided participants with automated feedback on their progress towards completing physical activity and nutrition goals, and the ability to interact with other participants on the website platform as a social support mechanism (Table 1). The IT-based intervention allowed participants to self-monitor physical activity and nutrition behaviours using either the website or mobile phone platform. A print-based comparison group was selected rather than a wait-list control group as it has been demonstrated that print-based interventions are effective [24,25]. Participants provided informed consent prior to participation and the study was approved by Central Queensland University, and the University of Western Sydney’s Human Research Ethics Committees. The study was registered with the Australian New Zealand Clinical Trials Registry (ACTRN12611000081910).

**Table 1:** Components of the ManUp intervention

**Major Component**	**Sub-component**	**Description**	**IT-based Intervention Arm**	**Print-based Intervention Arm**
**Educational Materials**				
	**Physical Activity**			
	What is physical activity	Description of	✓	✓
		- What is ‘physical activity’	✓	✓
		- The different intensities of physical activity	✓	✓
		- The physical activity guidelines for Australian adults		
	Benefits of physical activity	Summary of the health, social and economic benefits of physical activity participation	✓	✓
	Where to be active	Summary of the locations to be physically active in the study area, including parks, community facilities, commercial recreation centres.	✓	✓
	Getting started	Summary of the steps people should do to minimise risk when commencing a physical activity regime, including link to pre-exercise screening tool. Includes information on selecting the correct equipment for the activity, warming up, stretching, cooling down, progressively increasing activity, hydration and sun safety.	✓	✓
	Further information	URLs of other health promotion websites and resources for further information on physical activity	✓	✓
	**Healthy Eating**			
	What is healthy eating	Description of		
		- What is ‘healthy eating’	✓	✓
		- Where appropriate a serving size is defined	✓	✓
		- The healthy eating guidelines for Australian adults	✓	✓
	Benefits of healthy eating	Summary of the health benefits of healthy eating	✓	✓
	Where to eat healthily	Summary of the outlets that provide fresh food options in the study area	✓	✓
	Getting started	Summary of strategies to make choosing healthy eating a part of daily routines	✓	✓
	Further information	URL addresses of other health promotion websites and resources for further information on healthy eating	✓	✓
	**Body Weight**			
	What is a healthy body weight	Description of how a healthy body weight is defined using BMI and waist circumference	✓	✓
	Benefits of a healthy body weight	Summary of the health benefits of maintaining a healthy body weight	✓	✓
	How to achieve a healthy body weight	Summary of the strategies that can be used to maintain and/or achieve a healthy body weight	✓	✓
	Further information	URL addresses of other health promotion websites and resources to assist in maintaining and/or achieving a healthy body weight	✓	✓
**Self-monitoring**		- Ability to record progress towards completing any of the challenges	✓	✓
		- Ability to record body weight, height and waist circumference	✓	
		- Automatically generated summary of all data recorded	✓	
		- Ability to schedule an activity and receive text or email reminder	✓	
		- Visual summary of progress towards completing ManUp challenges	✓	✓
**Social-support**		- Ability to view ‘mates’ progress	✓	
		- Ability to comment on a the profile page of a mate	✓	
		- Ability to complete group based challenges	✓	
**ManUp challenges**		Light, Mid and Full Strength Physical Activity and Healthy Eating Challenges	✓	✓

### Participants, recruitment and group allocation

Eligible participants were males aged 35 to 54 years old who (1) owned a mobile telephone, (2) had access to the internet, (3) did not have a mobility impairment, (4) resided in the cities of Gladstone or Rockhampton (Queensland, Australia), and (5) were classified as low risk to commence an exercise regime [26]. Participants (n = 317) were recruited using a variety of techniques including advertisements via local newspapers, trading magazines, face-to-face information sessions with local businesses, and distribution of leaflets and posters to local businesses, medical clinics, and offices of allied health professionals. Rolling participant recruitment occurred from October 2010 to September 2011. Following initial screening for inclusion criteria, participants were randomly allocated to an intervention arm. Group assignment was conducted on a two-to-one ratio in favour of the IT-based intervention arm. Unequal group allocation was conducted to maximize the number of participants allocated to the intervention arm that is less frequently examined in male populations [19]. Randomization lists were generated using freely available software ( http://www.randomization.com). Participants were blinded to group allocation until baseline assessments were completed.

### ManUp intervention

The study was labeled “ManUp” to have men identify with the intervention and also to challenge men to take responsibility for their health. Similarities and differences in intervention components between the IT and print groups are summarized in Table 1 and reflected the ability of the IT-based intervention to include components related to automated feedback and participant interaction in an online environment. The ManUp intervention was designed on the basis of our review of physical activity interventions in males [12], formative research that identified the enablers and barriers that males faced in engaging in more physical activity and healthy eating [20] and the specific requirements for the IT-based components suggested by males [21].

Both intervention arms were designed to engage participants in making healthy changes to their physical activity and nutrition behaviours, and improve their health literacy about these behaviours. This was achieved by the provision of educational materials, increasing the frequency of participation in physical activity and healthy eating by the completion of a series of “challenges”, and self-monitoring progress towards the completion of each challenge. Participants were able to access their group’s intervention materials throughout the nine-month study period. Educational materials were specifically designed to be clear and uncomplicated in the presentation of the benefits of physical activity and healthy eating, the amount or type required to achieve health benefit, and how to achieve the amounts required for health benefits [12,20,21]. Educational materials were also designed to encourage males to change their health behaviours by recognizing how physical inactivity and poor nutrition can adversely affect health and by using this recognition as a stimulus to set goals to make positive changes to these behaviours [27]. The ManUp physical activity and healthy eating “challenges” were informed by Social Cognitive Theory and Self Regulation Theory, and developed to change target behaviours by having participants engage in goal setting and self-monitoring behaviours based on these challenges [28,29]. Goal setting and self-monitoring was operationalised by having participants select a challenge and record their progress towards completing the challenge.

### ManUp physical activity and healthy eating challenges

The “ManUp challenges” varied in duration and the amount of activity or healthy eating that males were asked to achieve. The challenges were designed to increase the overall levels of physical activity and healthy eating, rather than achieving any particular guideline for the behaviours. Variation in the duration and requirements for each challenge are described below. The challenge concept was adopted in ManUp based on our formative research [12,20,21]. Challenges were constructed to provide participants with specific, measureable, and time-based goals to achieve, which is consistent with established goal setting strategies [30], by specifying the weekly and total volume of activity across the duration of the challenge.

In total, six ManUp physical activity challenges (see Table 2) and a single ManUp healthy eating challenge were provided for participants to select from. For each challenge, participants could select from three different ‘strengths’ to participate in: light strength (three weeks), mid strength (six weeks) and full strength (12 weeks). The different strength challenges also varied in the weekly volume of physical activity and healthy eating to be completed and could be completed in any order. ManUp physical activity challenges included the activities of walking, cycling, swimming, running, strengthening, sport and recreation, and were selected based on recreation activities that Australian males frequently participate in [31]. The strengthening challenge included any resistance-based exercises such as free weights, machine weights, and body weight exercises. The sport and recreation challenge included any team-based activity or individual activity (i.e. soccer, football or group-based fitness class) not covered by the remaining five physical activity challenges.

**Table 2:** Description of the ManUp physical activity and healthy eating challenges

**Activity**	**Light Strength (3 weeks)**	**Mid Strength (6 weeks)**	**Full strength (12 weeks)**
Walking	1.5 hrs/week or	2.5 hrs/week or	3.5 hrs/week or
	7500 steps/day	10000 steps/day	12000 steps/day
Cycling	1 hr/week or	2 hrs/week	4 hrs/week
	25 km/week	or 50 km/week	or 100 km/week
Swimming	0.5 hr/week or	1 hr/week or	1.5 hrs/week or
	1 km/week	2 km/week	3 km/week
Running	0.5 hr/week or	1 hr/week or	2.0 hrs/week or
	5 km/week	10 km/week	20 km/week
Sport & Recreation	0.5 hr/week	1 hr/week	1.5 hrs/week
Strengthening	Set 8 exercises	Set 8 exercises	Set 8 exercises
	1 x set (8–10 reps)	2 x set (8–10 reps)	3 x set (8–10 reps)
	2 x/week	2 x/week	2 x/week
Healthy Eating	≥3 healthy eating goals/day	≥5 healthy eating goals/day	≥7 healthy eating goals/day

The ManUp healthy eating challenges were based on achieving a number of daily healthy eating goals: in total there were ten healthy eating goals that could be achieved. The goals were based on the Dietary Guidelines for Australian Adults, which promote eating a diverse diet that includes fruits, vegetables, grains, cereals, lean meat and fish while limiting the consumption of saturated fat, salt, alcohol and foods that contain added sugars [32]. The ManUp daily healthy eating goals were to: (1) eat two serves of fruit, (2) eat five serves of vegetables, (3) eat a serve of fish, (4) choose whole-grain bread instead of white bread, (5) choose low-fat dairy products, (6) have a soft drink- (soda-) free day, (7) have an alcohol-free day, (8) have an red-meat-free day, (9) have an unhealthy-snack-free day, and (10) have a fast-food-free day. To promote dietary diversity, participants were not constrained to pre-selecting any specific ManUp healthy eating goals for the duration of a healthy eating challenge. Rather, participants were encouraged to achieve any of the ten ManUp healthy eating goals on a daily basis and in order to complete a challenge successfully the number of healthy eating goals needed to be achieved varied by the strength of the challenge selected (Table 2).

Differences in challenge strengths were designed to cater for varying levels of initial physical activity and nutrition behaviours, to generate confidence to achieve a realistic target for behaviour change and to provide the opportunity to progressively increase changes to physical activity and dietary habits. Concise information on the ‘why’, ‘how’, and ‘where’ for each physical activity and healthy eating goal was provided to participants. The ‘why’ focused on the health, social, or economic benefits of engaging in the activity or achieving the healthy eating goal. The ‘how’ provided tips on how to integrate the activity or healthy eating goal into the daily routine to overcome barriers associated with participation. The ‘where’ provided participants with local information in the Gladstone and Rockhampton areas on where to engage in the activity or purchase the food or healthy alternatives to unhealthy dietary choices.

### Intervention arms

#### IT-based intervention arm

The IT-based intervention arm included access to the password-protected ManUp website. The website contained six sections that participants could navigate, including: My Profile, My Progress, My Mates, My Groups, My Weight, and Information Centre. The My Profile section summarized each participant’s progress in their current challenges, allowed participants to record their progress towards any current challenges and post personal updates to their profile, schedule future activities, and displayed their groups and a list of their ‘mates’ (online friends on the website). The My Progress page allowed participants to examine their progress graphs towards their current challenges. The website allowed participants to search for and view mates on the My Mates section of the website. The My Groups section allowed participants to create a group and view the progress of groups they were part of. The My Weight section provided information on the benefits of achieving a healthy weight and allowed participants to record their height, weight and waist circumference. This information was used to provide automatically generated classifications of health risk based on waist circumference and body mass index (BMI). The Information Centre provided participants with summaries of information related to physical activity and healthy eating, as described in Table 1, and also information about the physical activity and healthy eating challenges.

The IT-based intervention arm promoted social support, and friendly competition among male peers (group-based challenges) as these have been reported as effective strategies to promote engagement in physical activity and healthy eating [12]. Social support was operationalised by allowing men to ‘challenge’ their mates to compete with them to achieve a goal either in a one-on-one basis, or as part of a larger group. Men were also able to view the progress of their mates in any challenge they were enrolled in and to comment on their mates' My Profile page.

A mobile phone web application was developed as an additional tool to facilitate quick and convenient recording of progress towards the ManUp challenges, rather than as a platform to deliver educational material [21]. A mobile phone web application, rather than an “installed” application specific to a mobile platform (Apple, Android, Windows), was developed to maximize access to this additional self-monitoring tool. Any participant in the IT-based intervention arm who owned a mobile phone capable of accessing the internet had access to the mobile phone web application. The mobile phone web application allowed participants to enter their body weight, start a new ManUp physical activity or healthy eating challenge, record progress and view progress towards completing challenges. Both platforms were connected through protocols that enable data entry to be automatically synchronized between platforms on a frequently scheduled period.

#### Print-based intervention arm

Participants in the print-based group received a hard copy booklet that provided the same educational materials and ManUp challenges as those received by participants in the IT-based intervention. Participants in the print-based group were not provided with information on their peers in this group. The print-based booklet also included log sheets that could be used to monitor their progress and/or successful completion of any of the ManUp physical activity or healthy eating challenges.

#### Measures

All participants completed an online questionnaire at baseline and completed follow-up online questionnaires at 3 months and 9 months after they began the intervention. A randomly selected sub-sample (n = 91, 61 from IT-based intervention arm, 30 from print-based intervention arm) of participants also attended one of the trial centres (CQUniversity Gladstone or Rockhampton Campus) for an in-person assessment at each time point. During in-person assessments, participants completed the online survey, received an accelerometer for the objective measurement of physical activity, and had their height, weight and waist circumference measured by a trained research assistant.

#### Demographic characteristics

Participants self-reported the following demographic characteristics: age, employment status, level of employment, industry of employment, number of hours of work per week, household income, years of education, number of children (aged <18 years) living in the household, living situation, and presence of any chronic diseases diagnosed by a doctor.

#### Perceived health status

Participants reported on several aspects of perceived health status that have been identified as risk factors for health behaviours and chronic disease [16,33]. Two items were used to assess risk perception in relation to physical activity and body weight, consistent with previous research [16]. The items are “I believe that I am doing enough exercise/physical activity to achieve health benefits” and “I believe that my current body weight is a risk to my health.” Responses were measured on a five-point Likert type scale from strongly agree to strongly disagree [16]. The self-rated health item from the Healthy Days Module of the Behavioral Risk Factor Surveillance Survey (BRFSS) was used to assess self-rated health, and response options were excellent, very good, good, fair or poor [34].

#### Weight

All participants self-reported current height (cm), weight (kg), and waist circumference (cm) during the online survey. For those individuals selected for in-person assessments, these measures are also measured by project staff. During in-person assessments, participant height to the nearest 0.1 cm (PE087, Mentone Educational, Victoria, Australia), weight to the nearest 0.1 kg (Tanita BF-681, Tanita Corp., Tokyo, Japan) and waist circumference to the nearest 0.1 cm (Lufkin Executive W606PM) are measured at each assessment point, in triplicate. Participants are asked to wear light clothing and to remove shoes prior to the assessment. Waist circumference is measured horizontally at the umbilicus, after normal expiration, by a trained research assistant.

#### Activity related behaviours

Physical activity was assessed in all participants using the Active Australia Questionnaire, which assesses the frequency and duration of transport and recreational walking, moderate and vigorous intensity physical activity [35]. The Active Australia Questionnaire has demonstrated acceptable levels of test re-test reliability and validity in the Australian adult population, and has been identified as a useful measure to detect intervention-related change in physical activity behaviours [36-39]. Participants (n=91) who selected to attend in-person measurement sessions were fitted with an ActiGraph GT3X (ActiGraph, Pensacola, FL) accelerometer to provide objective measures of activity behaviours over a five-day period. Participants were instructed to wear the ActiGraph, mounted on an elastic belt around the waist with the unit positioned over the right hip during all waking, non-contact activities (thus excluding activities like rugby league or rugby union) and non-water-based activities. The ActiGraph monitors were set to record steps, inclination, and acceleration counts in tri-axial mode, using a 10-second epoch. Accelerometer data was analysed using the MeterPlus program [40] in 10 second epochs using previously reported cut-points for sedentary, light and moderate-to-vigorous intensity physical activity [41]. Non wear time was assessed using a minimum of 60 minutes of consecutive zero counts allowing up to a 2 minute tolerance of non-zero counts. A minimum of 10 hours per day of wear time on at least four days was required to be included in analysis.

Self-reported duration of sitting in occupational settings over the previous seven days was assessed using two items. Adapted from an existing measure of occupational sitting [5,42], the first item asked participants to report the amount of time sitting at work during meetings, lunch and at their desk. Using the same recall period the second item asked participants to report the amount of time spent driving at work. Both items asked participants to report the duration of sitting in hours and minutes. Duration of sitting in leisure time was assessed using items adapted from a existing measure of nine leisure-time sedentary behaviours with demonstrated acceptable test-retest reliability [43]. The sedentary behaviours that were assessed included computer use, hobbies, television viewing, sitting and socializing, reading, sitting or lying down while listening to music, talking on the telephone, going for a recreational drive, and relaxing, thinking and resting [43]. These items were modified to explicitly ask about time spent sitting while performing each behaviour, instead of time spent engaged in each behaviour.

#### Nutrition behaviours

Nutrition behaviours related to the healthy eating guidelines for Australian adults [32] were assessed using 19 items adapted from the National Health Survey Australia and the Monitoring Food Habits Questionnaire [44,45]. The number of daily serves of fruit and vegetables usually consumed in the previous week was assessed using two separate items, based on those used in the National Health Survey [44]; possible response options were: one serve or less; two to three serves; four to five serves; six or more; and don’t consume this food. The number of times in the previous week that red meat, fish, meat products (sausages, salami, meat pies, etc.), cooked cereals, bread, soft drink, chips, takeaway foods, and sweet or savoury foods were consumed was assessed using a nine-point scale from rarely/never to more than seven times per week, each item also included a “don’t consume this food” response option. The volume of milk consumed each day and the type of milk (whole milk, reduced fat, soy milk, condensed milk) was assessed using two separate items [44].

#### Health literacy

Health literacy can be defined as the acquisition of a set of skills and knowledge that can be applied to change behaviours and improve health [22,46]. The five physical activity awareness items from the Active Australia Questionnaire were used to assess health literacy related to physical activity [35]. These items assess awareness related to the health benefits of physical activity that include the minimum amount of physical activity required for health benefit, the appropriate intensity of physical activity to achieve benefit, and the pattern in which physical activity can be accumulated to receive health benefits [35]. The 28-item Nutritional Literacy Survey was used to assess health literacy related to healthy eating [47]. This instrument presents sentences to the respondent that contain one or more words removed from the sentence, the respondent is provided with four possible response options and asked to select the response that best fits the sentence [47]. Topics covered include food types that promote heart health, fat and cholesterol contents of food, and portion size [47].

#### IT platform usage

Both the website and mobile platforms allow monitoring of the number of times a participant has logged in, the number and date of entries made, and the number of challenges created and completed. The website platform also permits monitoring of which educational resource pages have been viewed by participants. These measures describe the level of participant engagement with the intervention components.

#### Sample size

The study was powered to detect a 60-minute increase in moderate-to-vigorous intensity physical activity using a 0.05 alpha, with a power level of 90%. Based on these factors, it was estimated that 197 participants would be required [48]. Typical dropout rates in IT based interventions are approximately 30% [16]. Given the difficulty in engaging and retaining the target population (middle aged males) the estimated sample size was increased to account for a 45% dropout rate. Methods described by Hsieh to account for the loss of power associated with the 2:1 group allocation [49], the estimated sample size was further inflated using a variance inflation factor of (VIF = 1.125) resulting in a total estimated sample size of 321; 107 allocated to the print-based group and 214 allocated to the IT-based group.

#### Analysis

Comparison of sample baseline characteristics between intervention arms were examined using chi-square tests for proportions, and either linear, gamma or Poisson generalized linear models for continuous or count data. Examination of change in outcomes will be based on group allocation and the intention-to-treat principle. Linear mixed models and generalized estimating equations will be used to compare intervention groups across time points [50]. Statistical significance was set at a *p*-value of 0.05.

#### Baseline characteristics of the sample

**Figure 1:**
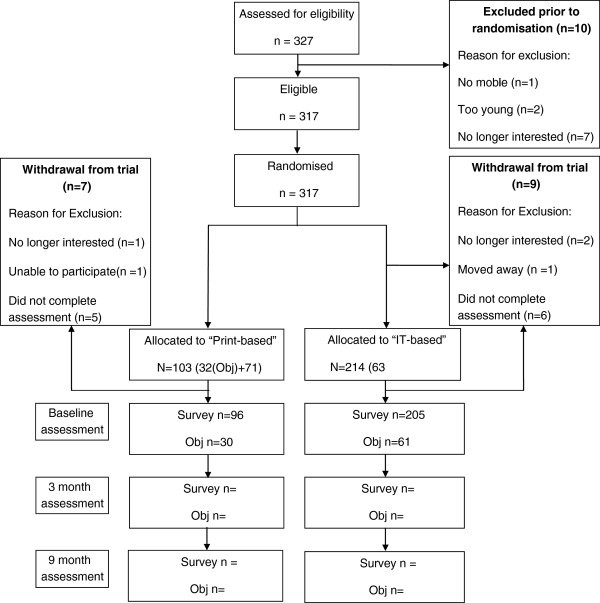
**Flowchart describing the progress of participants through trial phases.** Obj – Objective or ‘in person’ measurement completed with randomly selected sub-sample of participants.

A total of 327 males contacted the research team about participation in the study, 10 males withdrew from the study prior to randomization to an intervention arm (Figure 1). The primary reason for not continuing participation in the study was being no longer interested in participation after having details of the study explained to them. Following allocation to an intervention arm, nine participants in the IT-based intervention arm and seven participants in the print-based intervention arm did not complete a baseline assessment and were withdrawn from the trial (Figure 1). Participants were asked how they learned about the existence of the ManUp project (for which they could report multiple methods); responses were classified into the following categories 1) project specific advertisements and promotional materials (n = 152), 2) word of mouth (n = 87), 3) information provided at their workplace (n = 75), 4) direct contact with project staff (n = 49), and 5) not specified (n = 1).

Participant socio-demographic characteristics at baseline are provided in Table 3. The average age of IT-based and print-based intervention arms was similar (44.2 vs. 43.8 *p* = .656). In both intervention arms, the majority of participants were classified as working in professional occupations; there were no significant differences between intervention arms in the proportion of participants employed in professional, white collar, blue collar or other occupation categories (*p* = .639). All participants owned a mobile phone, however only 151 (73.2%) of those in the IT-based intervention arm owned a mobile phone that was capable of accessing the internet. The average BMI (calculated from self-reported height and weight) was 30.9 and 30.4 in the IT-based and print-based intervention arms respectively (*p* = .434), when classifying BMI in established BMI categories, over 85% of participants in both intervention arms were classified as either overweight or obese (Table 3). The IT-based and print-based intervention arms did not significantly differ on average for the in-person measured BMI (29.8 vs. 30.2, *p* = .712) and over 80% of participants were classified as overweight or obese when using in-person measured BMI. There were no significant between-group differences for any socio-demographic variable examined in Table 3.

**Table 3:** Baseline comparisons of socio-demographic and anthropometric characteristics between ManUp intervention groups (n = 301)

	**Print-based**		**IT-based**		
**Variable**	**N**	**%, M (SE)**	**N**	**%, M (SE)**	**p**
Age	96	43.8 (.6)	205	44.2 (.4)	.656
*Occupational Classification*					
% Professional	52	54.2%	118	57.6%	
% White Collar	8	8.3%	16	7.8%	
% Blue Collar	23	24.0%	37	18.0%	
% Other^b^	13	13.5%	34	16.6%	.639
Education Level					
% Secondary School or Less	20	20.8%	45	22.0%	
% TAFE	25	26.0%	61	29.8%	
% University	51	53.1%	99	48.3%	.719
Self-Report Weight	96	96.3 (2.0)	205	98.3 (1.4)	.389
Self-Reported BMI	96	30.4 (.5)	205	30.9 (.4)	.434
Self-Reported BMI Classification					
*% Healthy Weight*	12	12.8%	19	9.3%	
*% Overweight*	40	42.6%	85	41.5%	
*% Obese*	42	44.7%	101	49.3%	.588
Self-Reported Waist Circumference	96	98.9 (2.0)	205	99.6 (1.4)	.782
Self-Reported Waist Circumference Classification					
*% Healthy*	33	34.4%	59	28.8%	
*% Risky*	28	29.2%	50	24.4%	
*% High Risk*	35	36.5%	96	46.8%	.239
Self-Rated Health Classification					
*% Fair or Poor*	25	26.0%	73	35.6%	
*% Good*	37	38.5%	83	40.5%	
*% Very Good or Excellent*	34	35.4%	49	23.9%	.080
Measured Weight^a^	30	94.7 (3.1)	61	93.3 (2.2)	.719
Measured BMI^a^	30	30.2 (.9)	61	29.8 (.6)	.712
Measured BMI Classification^a^					
*% Healthy Weight*	3	10.0%	10	16.4%	
*% Overweight*	14	46.7%	26	42.6%	
*% Obese*	13	43.3%	25	41.0%	.713
Measured Waist Circumference^a^	30	101.9 (2.3)	61	102.0 (1.6)	.968
Measured Waist Circumference Classification^a^					
*% Healthy*	6	20.0%	15	24.6%	
*% Risky*	11	36.7%	19	31.1%	
*% High Risk*	13	43.3%	27	44.3%	.830

Note. ^a^ A randomly selected sub-sample (n = 91) of participants completed in person assessments of height, weight, waist circumference and physical activity (ActiGraph) these are referred to as “measured” outcomes.

^b^Other category includes participants who were retired, students, unemployed or a pensioner.

Table 4 shows that there were no significant between-group differences in: self-reported minutes or sessions of physical activity; sitting during leisure time; sitting at work; or minutes of objectively determined minutes of sedentary, light and moderate-to-vigorous intensity physical activity. When examining physical activity literacy, the only significant between-group difference was that a lower proportion of participants in the IT-based intervention arm agreed that 30 minutes each day is enough to improve health compared to the print-based intervention arm (70.2% vs. 82.3%, *p* = .026). No significant differences were observed between the IT-based intervention arm and the print-based intervention arm on the proportion of participants who reported eating at least two serves of fruit per day (38.5% vs. 35.4%, *p* = .602) and five or more serves of vegetables per day (5.9% vs. 5.2%, *p* = .821). The print-based intervention arm consumed a higher number of serves of red meat compared to the IT-based intervention arm (*p* = .017). No other significant differences were observed in nutrition behaviours or levels of nutrition literacy between the intervention groups.

**Table 4:** **Baseline comparisons of physical activity and dietary behaviours between ManUp intervention groups (n = 301)**^**a**^

	**Print-based**		**IT-based**		
	**N**	**%, M (SE) or median(1**^**st**^**and 3**^**rd**^**quartile)**	**N**	**%, M (SE) or median(1**^**st**^**and 3**^**rd**^**quartile)**	**p**
**Self-report physical activity behaviours**					
Weekly minutes of physical activity	96	277.9 (34.0)	205	286.1 (23.3)	.843
Weekly session of physical activity	96	4 (1,8)	205	4 (1,7)	.892
Physical activity classification					
*% None*	19	19.8%	39	19.0%	
*% Insufficient*	36	37.5%	88	42.9%	
*% Sufficient*	41	42.7%	78	38.0%	.655
Daily minutes of sitting outside of work	96	520.1 (27.2)	205	492.9 (18.6)	.409
Daily minutes of sitting at work	96	411.0 (26.1)	205	452.4 (17.9)	.191
**Objective Physical Activity Behaviours**^**a**^					
Daily minutes of sedentary behaviour	25	631.0 (18.5)	52	670.6 (12.8)	.079
Daily minutes of light intensity physical activity	25	184.9 (8.4)	52	182.1 (5.8)	.786
Daily minutes of moderate-to-vigorous intensity physical activity	25	46.7 (4.9)	52	44.8 (3.2)	.751
**Physical Activity Literacy (% Agree)**					
> 30 minutes/day improves health	79	82.3%	144	70.2%	.026
30 minutes brisk walking improves health	79	82.3%	153	74.6%	.141
20 minutes of Vigorous activity 3 times a week is essential	54	56.3%	139	67.8%	.051
10 minute blocks of activity are okay	52	54.2%	106	51.7%	.690
Moderate activity can improve health	87	90.6%	177	86.3%	.291
**Dietary Habits**					
# Serves vegetables/day	96	2 (1,2)	205	2 (1,3)	.793
# Serves fruit/day	96	1 (1,2)	205	1 (1,2)	.735
# Serves of red meat last week	96	4 (3,6)	205	4 (3,5)	.017
# Soft drinks last week	96	2 (0,3)	205	1 (0,4)	.783
# Times fast food/takeaway	96	2 (0,2.75)	205	(1 (.5,2)	.339
Ave. Serves of Alcohol on a drinking day	96	2.78 (.3)	205	2.68 (.2)	.761
# Days of Harmful Drinking					
% 0 days	45	46.9%	102	49.8%	
% 1–2 days	29	30.2%	60	29.3%	
% ≥3 days	22	22.9%	43	21.0%	.885
Bread Type					
*% White*	40	42.6%	62	31.3%	
*% Grain*	53	56.4%	133	67.2%	
*% Don’t Eat Bread*	1	1.1%	3	1.5%	.168
Milk Type					
*% Full Cream*	37	38.5%	80	39.0%	
*% Low Fat*	50	52.1%	102	49.8%	
*% Soy/Condensed*	2	2.1%	5	2.4%	
*% Don’t Drink Milk*	2	2.1%	5	2.4%	
*% Other*	5	5.2%	13	6.3%	.991
Nutritional Literacy	96	25 (24, 26)	205	26 (24, 27)	.656

Note: ^a^ A randomly selected sub-sample (n = 91) of participants completed in person assessments of height, weight, waist circumference and physical activity (ActiGraph). Variation in sample size is due to participants not fulfilling wear time criteria and were excluded from analysis of objective measures of physical activity.

## Discussion

This paper describes the intervention design, study protocol and baseline characteristics of a sample of middle aged males who took part in the ManUp Study. This study was designed in an attempt to address some of the key issues associated with conducting research in the middle-aged male population. These issues include specifically designing the intervention with the needs and preferences of males in mind to increase intervention appeal, and focusing on behavioural risk factors most relevant to men. The study protocol will allow the efficacy of an IT-based delivery of the intervention to be compared to a print-based delivery mode. If the IT-based delivery mode is found to be efficacious, it will provide the foundation for similar interventions that take advantage of the increased reach of IT-based interventions to be developed for this population group. Within the IT-based intervention arm, it will be possible to determine the presence of any relationships between engagement with the platform and change in outcomes. This will provide much needed information in this area as reviews of such interventions have identified that a low proportion of males engage in these interventions [12,19]. The ManUp Study will also collect information on the components of the IT-based intervention that are utilized, and this information can be used to refine the strategies adopted in future IT-based interventions by focusing attention on those that participants use most frequently.

Males are frequently reported to have high levels of anthropometric and behavioural risk factors of poor health, and this is the underlying rationale for the current study [2,3,8]. Baseline characteristics of the ManUp Study participants indicate, that like many males, they are generally overweight or obese, have poor nutrition behaviours and have a low level of total physical activity. Over 85% and 80% of participants are classified as overweight or obese when using self-reported and measured BMI respectively; this is higher than levels of self-reported overweight or obesity in Queensland and Australian adult males [3,8,51]. The proportion of participants who self-reported physical activity at a level sufficient to meet guidelines in print- and IT-based intervention arms (42.7% and 38.0%) is lower than that observed in population-based samples covering similar geographical areas (52.8%) [6]. Furthermore, in comparison to other available data [52], the current sample spends more time in sedentary behaviour and less time in light intensity physical activity. The average nutrition behaviours of the study sample were below that recommended for Australian adults, and the proportion of the sample who achieved the minimum guidelines for fruit and vegetables was lower than that reported for other Australian males [8].

Levels of physical activity literacy in the current sample were lower than those previously reported in Australian males [53], particularly for awareness that accumulating physical activity in 10-minute blocks can still provide health benefits. Over a decade has passed and significant financial investment has been directed toward the promotion of physical activity in Australia and the levels of physical activity literacy in the current sample are lower than those reported in 1999 [53]. While this is concerning from a health promotion perspective, it also provides a unique opportunity to intervene and attempt to address the issue in the current sample of participants to inform subsequent promotion efforts. In contrast, levels of nutrition literacy were relatively high with the median score of both intervention groups close to the maximum level and comparable to levels reported in the sample the instrument was developed in [47]. This is a positive finding in the current study, and somewhat unexpected given that males in our formative study reported confusion around nutritional messages [20].

Subsequent phases of the ManUp Study will evaluate changes in physical activity, nutrition behaviours, and health literacy of those topics over the intervention period, and will compare the efficacy of the two intervention arms to change these outcomes.

## Competing interests

The authors declare that they have no competing interests.

## Authors’ contributions

MJD drafted the manuscript and completed statistical analysis. AM, RT, MK and HD developed the IT platform. MJD, CV, CH and ME were responsible for participant recruitment and overseeing study implementation. All authors contributed to the design of the overall study, development of intervention materials, read, edited and approved the final manuscript.

## Pre-publication history

The pre-publication history for this paper can be accessed here:

http://www.biomedcentral.com/1471-2458/12/656/prepub
